# Analysis of miRNA profiles identified miR-196a as a crucial mediator of aberrant PI3K/AKT signaling in lung cancer cells

**DOI:** 10.18632/oncotarget.13432

**Published:** 2016-11-17

**Authors:** Ilaria Guerriero, Daniela D’Angelo, Pierlorenzo Pallante, Mafalda Santos, Marianna Scrima, Donatella Malanga, Carmela De Marco, Alessandro Weisz, Carmelo Laudanna, Michele Ceccarelli, Geppino Falco, Antonia Rizzuto, Giuseppe Viglietto

**Affiliations:** ^1^ Biogems.c.ar.l., Istituto di Ricerche Genetiche “Gaetano Salvatore”, Ariano Irpino (Avellino), Italy; ^2^ Istituto per l’Endocrinologia e l’Oncologia Sperimentale, IEOS-CNR, c/o Dipartimento di Medicina Molecolare e Biotecnologie Mediche, Università “Federico II” of Napoli, Napoli, Italy; ^3^ Dipartimento di Medicina Sperimentale e Clinica, Università Magna Graecia of Catanzaro, Italy; ^4^ Laboratorio di Medicina Molecolare e Genomica, Dipartimentodi Medicina e Chirurgia, Università di Salerno, Baronissi, Italy; ^5^ Department of Biological and Environmental Studies, Università del Sannio, Benevento, Italy; ^6^ Dipartimento di Biologia, Università degli Studi di Napoli Federico II, Complesso Universitario Monte S.Angelo, Napoli; ^7^ Dipartimento di Scienze Mediche e Chirurgiche, Università Magna Graecia of Catanzaro, Italy

**Keywords:** NSCLC, PI3K/AKT, microRNA, miR-196a

## Abstract

Hyperactivation of the PI3K/AKT pathway is observed in most human cancer including lung carcinomas. Here we have investigated the role of miRNAs as downstream targets of activated PI3K/AKT signaling in Non Small Cell Lung Cancer (NSCLC). To this aim, miRNA profiling was performed in human lung epithelial cells (BEAS-2B) expressing active AKT1 (BEAS-AKT1-E17K), active PI3KCA (BEAS-PIK3CA-E545K) or with silenced PTEN (BEAS-shPTEN).

Twenty-four differentially expressed miRNAs common to BEAS-AKT1-E17K, BEAS-PIK3CA-E545K and BEAS-shPTEN cells were identified through this analysis, with miR-196a being the most consistently up-regulated miRNA. Interestingly, miR-196a was significantly overexpressed also in human NSCLC-derived cell lines (n=11) and primary lung cancer samples (n=28).

By manipulating the expression of miR-196a in BEAS-2B and NCI-H460 cells, we obtained compelling evidence that this miRNA acts downstream the PI3K/AKT pathway, mediating some of the proliferative, pro-migratory and tumorigenic activity that this pathway exerts in lung epithelial cells, possibly through the regulation of FoxO1, CDKN1B (hereafter p27) and HOXA9.

## INTRODUCTION

Lung cancer is the most frequent cause of cancer-related deaths worldwide that is divided in two main groups: small-cell lung cancer (SCLC, accounting approximately 20%) and non-small-cell lung cancer (NSCLC, approximately 80%) [1, 2]. Despite advances in early detection and standard treatment, NSCLC patients often have poor prognosis, with five-year survival rate less than 15% [3, 4].

Recent studies have shown that the phosphatidylinositol 3-kinase (PI3K) signaling cascade is frequently activated in human cancer [5–7]. PI3K signaling regulates multiple cellular processes that are critical for tumorigenesis that include cell proliferation, migration, apoptosis, glucose metabolism and angiogenesis [8, 9].

In NSCLC, aberrant activation of the PI3K/AKT pathway has been shown to contribute to both cancer initiation and progression [10–13]. Hyperactivation of AKT is detected in most NSCLC cell lines [14–16] and in 30–75% NSCLCs [17–21], promoting resistance to chemo- and radiotherapy [15].

The end-point of the PI3K pathway is AKT, a small family of serine/threonine protein kinases. AKT is activated by recruitment to cell membrane via binding of its PH domain to 3′-phosphorylated phosphatidylinositols generated by PI3K and subsequent phosphorylation at T308 and S473 [9, 14–27]. Conversely, the lipid phosphatase PTEN attenuates AKT activation by dephosphorylating the 3′ position of phosphatidylinositols [28].

Once activated, AKT phosphorylates a number of downstream substrates that include Bad, Bim, procaspase-9, IκKalpha, the forkhead family of transcription factors FoxO1, FoxO3a, GSK-3β, the ubiquitin ligases MDM2 and SKP2, the CDK inhibitors p21 and p27 and others [29].

However, whereas the effects of AKT activation on mRNAs and proteins in the cell are known fairly well, so far the role of miRNAs as downstream targets of activated PI3K/AKT signaling is still poorly defined.

MiRNAs are small, conserved, noncoding RNAs of 18–25 nucleotides in length that act as negative regulators of many genes involved in cell development, differentiation, proliferation, survival and death [30]. For this reason, miRNAs are important players in the pathogenesis of cancer, acting either as oncogenes or tumor suppressor genes [31–33]. Expression of miRNAs is systematically altered in several cancers and the rearrangement of many genes encoding miRNAs has been associated to multiple cancers [34]. NSCLC samples frequently present alteration of the expression of miRNAs [35], and a plasmatic miRNA profile predicts the presence of an active disease [36]. Interestingly, miRNAs may act either as oncogenes or tumor suppressor genes and are involved in cancer initiation, dissemination and response to therapy [37–44].

To determine the role of miRNAs in lung cancer driven by aberrant PI3K/AKT signaling, we have examined changes in miRNA levels that are induced by alterations of this pathway through different mutations that are observed in human NSCLC [11], including a gain-of-function mutant of PIK3CA (E545K), a gain-of-function mutant of AKT1 (E17K) and the loss of PTEN expression [12, 13, 45, 46]. Here we report that miR-196a is modulated by mutant PI3K, mutant AKT1 and by loss of PTEN, and represent a pivotal mediator of proliferation, migration/invasion and tumorigenicity elicited by aberrant activation of PI3K/AKT pathway.

## RESULTS

### Identification and validation of PI3K/AKT-dependent miRNA expression signature

**Figure 1:**
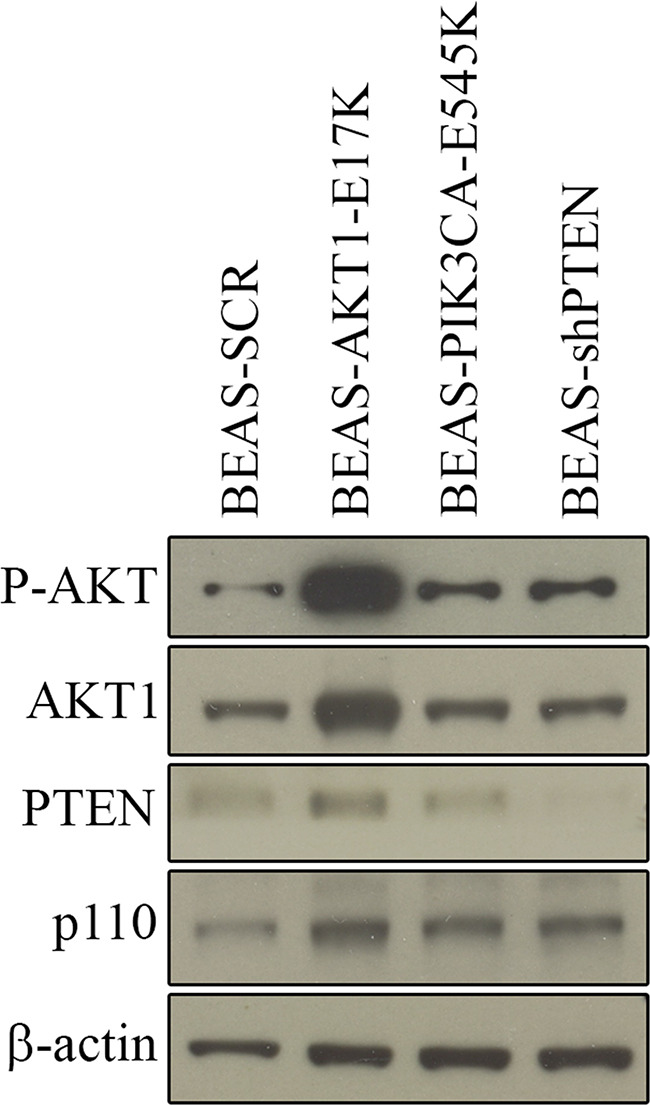
Expression of AKT1-E17K, PIK3CA-E545K, PTEN in BEAS-2B cells and derivatives Immunoblot analysis of BEAS-2B cells and derivatives for the expression of the phosphorylation of AKT and for the expression of AKT1, PTEN, p110. ß-actin was used as loaded control.

The aim of this study was to identify a miRNA signature of aberrant PI3K/AKT signaling in NSCLC cells. To this aim, we have genetically modified bronchial epithelial cells that recapitulate the most frequent alterations within the PI3K/AKT pathway. Immortalized, non-tumorigenic human bronchial epithelial cells (BEAS-2B) were engineered to express gain-of-function mutations of PIK3CA (PIK3CA-E545K), of AKT1 (AKT1-E17K) or were silenced for PTEN (BEAS-AKT1-E17K, BEAS-PIK3CA-E545K and BEAS-shPTEN, respectively) as described in previous work [47, 48]. However, to perform the experiments indicated below we infected BEAS-2B cells *de novo* so that both parental and derivative cells could be used at early passages. The presence of exogenous mutant PIK3CA, mutant AKT1 or of endogenous wild-type PTEN proteins in transduced cells as well as the activation of PI3K/AKT signaling was determined by immunoblot and are shown in Figure 1.

**Figure 2:**
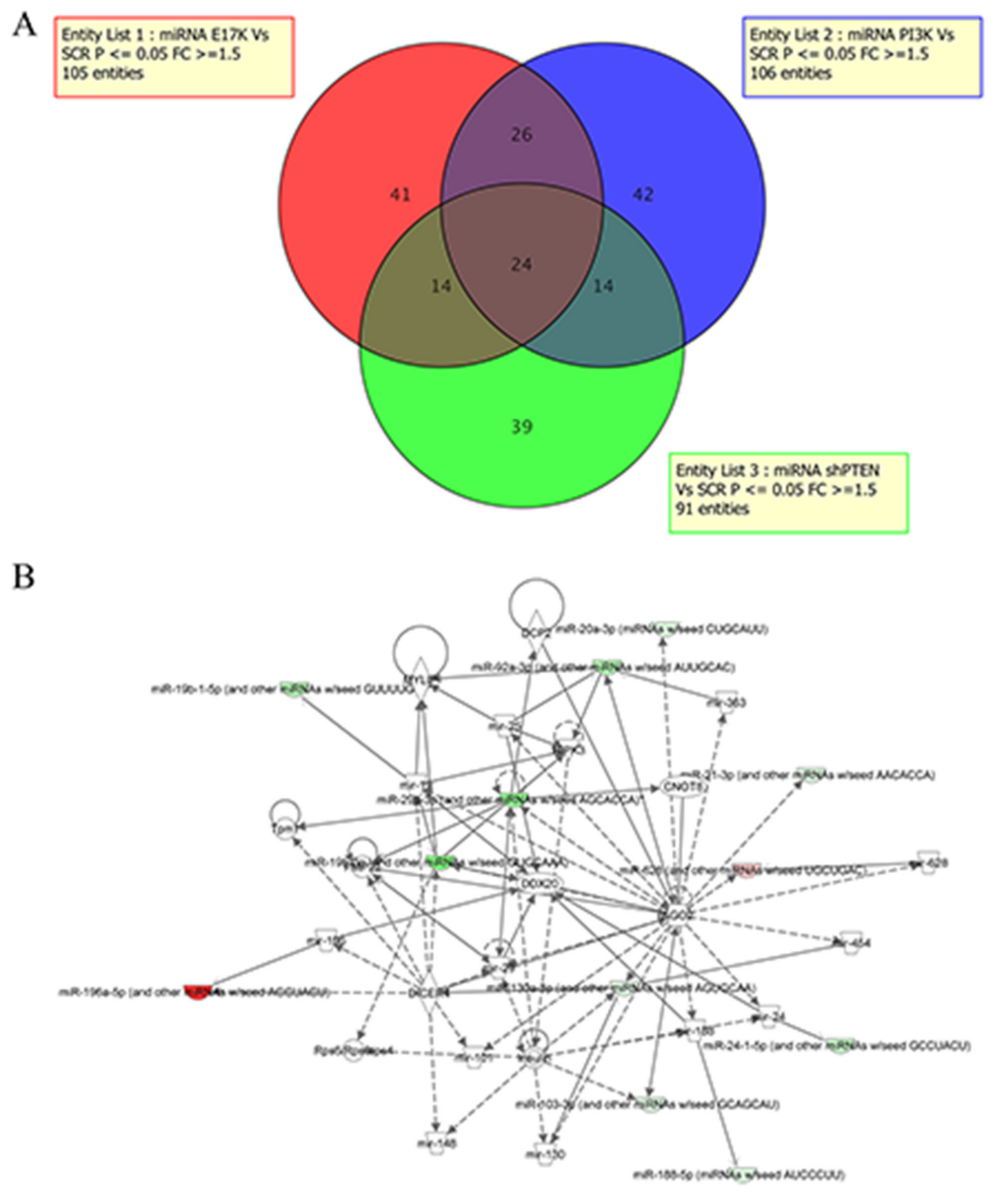
MiRNA profiling of BEAS-2B cells and derivatives **A**. Venn diagram of DEMs in BEAS-AKT1-E17K, BEAS-PIK3CA-E545K and BEAS-shPTEN. **B**. Network analysis was performed to provide a graphical representation of miRNAs and genes having known biological relationships. Green icons indicate down-regulated miRNAs and genes and red icons indicates up-regulated miRNAs.

MiRNAs targets of constitutive signaling of PI3K/AKT in lung cancer cells were identified by miRNA profiling of BEAS-2B cells and derivatives. Expression values of miRNAs obtained were filtered for fold change >1.5 and subjected to t-test (p-value cut-off: 0.05) with Benjamini-Hochberg (B–H) FDR correction [49]. Analysis of the results allowed to identify 105 differentially expressed miRNAs (DEMs) in cells expressing mutant AKT1, comprising 42 up-regulated and 63 down-regulated, 106 DEMs in cells expressing mutant PIK3CA, 54 up-regulated and 52 down-regulated, and 91 DEMs in cells silenced for PTEN, 45 up-regulated and 46 down-regulated (Figure 2A). The complete microarray data for all probe sets with the respective normalized values will be available at ArrayExpress (E-MTAB-4263) and are provided in additional files (Supplementary Tables S1–S3).

**Table 1:** The most significant DEMs modulated in BEAS-2B cells that are common to mutant AKT1, PIK3CA or PTEN loss with their relative fold change

Up-regulated miRNAs (Fold change)			
miRNA ID	BEAS-AKT1-E17K(fold change)	BEAS-PIK3CA-E545K(fold change)	BEAS-shPTEN(foldchange)
hsa-miR-196a	8.3	3.1	2.7
hsa-miR-203	6.5	1.9	7.4
hsa-miR-187	2.6	3.2	1.9
hsa-miR-628-5p	2.3	1.7	2.3
HS_243.1	2.3	1.7	3.6
HS_284.1	1.9	1.8	2.5
**Down-regulated miRNAs (Fold change)**			
**miRNA ID**	**BEAS-AKT1-E17K(fold change)**	**BEAS-PIK3CA-E545K(fold change)**	**BEAS-shPTEN(foldchange)**
hsa-miR-33a	−16.1	−7.2	−8.8
hsa-miR-29c	−7.4	−3.6	−4.4
hsa-miR-219-5p	−5.2	−2	−3.6
hsa-miR-19b-1*	−5.1	−2	−3.8
hsa-miR-16-1*	−4.6	−2.9	−3.8
hsa-miR-424	−4.2	−2	−4.5
hsa-miR-24-1*	−3.9	−2.3	−4.1
hsa-miR-542-3p	−2.9	−2.2	−3.8
hsa-miR-29b	−2.6	−2.1	−3.3
hsa-miR-301a	−2.4	−1.8	−2
hsa-miR-107	−2	−1.7	−2.2
hsa-miR-101	−1.8	−1.9	−1.8
hsa-miR-188-5p	−1.7	−1.6	−1.8

Based on the 3 lists of DEMs, we focused our attention on the miRNAs whose expression was influenced specifically by the oncogenic alteration of AKT1, PIK3CA or PTEN, and, alternatively, on those commonly deregulated by two or three of the above-mentioned alterations. We thereby found that 41/1145 DEMs analyzed (3.5%) were modulated by mutant AKT1 (15 up-regulated, 26 down-regulated), 42 DEMs analyzed (3.6%) were modulated by mutant PIK3CA (25 up-regulated, 17 down-regulated) and 39 DEMs analyzed (3.4%) were modulated by PTEN loss (22 up-regulated, 17 down-regulated; listed in Supplementary Tables S4–S6).

Once we have identified miRNAs regulated by activated AKT1 or PIK3CA, as well as those modulated by PTEN silencing, we proceeded to match the lists of DEMs in order to identify the miRNAs that, in lung cells, were common to the alterations of AKT1 and PIK3CA, AKT1 and PTEN and/or PIK3CA and PTEN, respectively, or common to all 3 genetic alterations. These DEMs are more likely to be the most relevant mediators of aberrant PI3K/AKT signaling in transformed bronchial epithelial cells. As shown in the Venn diagrams of Figure 2A, 14 DEMs (7 up-regulated, 5 down-regulated and 2 discordant) were common to BEAS-PIK3CA-E545K and BEAS-shPTEN, 26 (12 up-regulated, 12 down-regulated and 2 discordant) to BEAS-AKT1-E17K and BEAS-PIK3CA-E545K cells, 14 (6 up-regulated, 7 down-regulated and 1 discordant) to BEAS-AKT1-E17K and BEAS-shPTEN cells and, finally, 24 (6 up-regulated, 13 down-regulated and 5 discordant) to all three cell lines studied. This indicates that, altogether, aberrant PTEN/PI3K/AKT signaling regulated the expression of 200/1145 miRNAs (17.5%), though only 24 were common to all three alterations (2%).

Among the DEMs that were common to BEAS-AKT1-E17K, BEAS-PIK3CA-E545K and BEAS-shPTEN cells miR-203, miR-196a and miR-187 showed the highest fold changes. Conversely, among down-regulated miRNAs common to all three aberrations, miR-33a, miR-29c and miR-219-5p showed the highest fold changes. See Table 1 for a list of the most representative DEMs common to all three alterations with the corresponding fold-changes.

### Categorization of DEMs regulated by PI3K/AKT signaling in human lung epithelial cellsby Ingenuity Pathway Analysis

Subsequently, we investigated by Ingenuity Pathway Analysis (Ingenuity® Systems, http://www.ingenuity.com, IPA) the functional significance of changes in the miRNA expression, and performed a categorization of these datasets into bio-functions and networks as indicated (Supplementary Figures S1–S2). The function “Reproductive System Disease” was the most frequent for the DEMs common to BEAS-AKT1-E17K, BEAS-PIK3CA-E545K and BEAS-shPTEN, and was associated with 16 miRNAs, followed by “Cancer” (14 miRNAs), “Gastrointestinal Disease” (12 miRNAs), “Endocrine System Disorders” (10 miRNAs), and “Hepatic System Disease” (8 miRNAs), respectively.

We then analyzed the different categories associated with the bio-function “Cancer” and found different terms referring to lung cancer including “Lung squamous cell carcinoma” (2 molecules), “Small cell lung cancer” (1 molecule), “Non small cell lung cancer” (2 molecules) (Supplementary Table S7). The DEMs identified within these functions were miR-16-1*/miR-16-5pand miR-203 for the function “Lung squamous cell carcinoma”, miR-19b for the function “Small cell lung cancer” and miR-16-5p and miR-196a for the function “Non small cell lung cancer”, indicating that these DEMs as the most relevant for lung cancer. In particular, aberrant expression of miR-196a has been frequently reported in various cancers including NSCLC [50–53] and it represents a major regulator of migration in NSCLC cells [54].

For further studies we chose miR-196a on the basis of the following considerations. MiR-196a was the DEM with the highest average fold change, being >2 in all three BEAS-AKT1-E17K, BEAS-PIK3CA-E545K and BEAS-shPTEN cell lines. MiR-196a also turned out to be the only DEM present in relevant networks (Figure 2B, Network 2) by IPA analysis where it was the most highly up-regulated among the DEMs of Network 2 (Supplementary Figure S2).

### Analysis of miR-196a expression in NSCLC-derived cell lines and primary tumors

**Figure 3:**
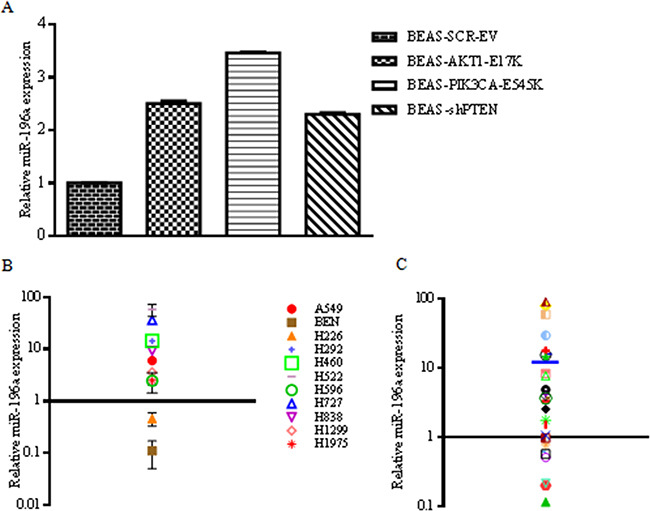
Expression of miR-196a in lung cancer-derived cell lines and primary tumors **A**. Relative mRNA expression of miR-196a by qRT-PCR in control BEAS-2B and derivatives. **B**. Expression of miR-196a in human NSCLC cell lines by qRT-PCR. Data are reported with respect to normal human bronchial epithelial cells (NHBE) whose value has been arbitrarily established as 1 and marked with a thick black line. Fold change values on y-axis are reported as log10. **C**. Expression of miR-196a in 28 human primary NSCLC samples by qRT-PCR. Normal human lung tissues (n=6) were used as controls to obtain an average expression value of miR-196a in normal lung that has been arbitrarily established as 1 and marked with a thick black line. Mean fold-change value (14) is shown as a blue line. Fold change values on y-axis are reported as log10.

First, we confirmed expression of miR-196a in BEAS-2B cells and derivatives by using quantitative RT-PCR (Figure 3A). Subsequently, we analyzed the expression of miR-196a in NSCLC cell lines and primary tumors. Figure 3B shows the levels of miR-196a in 11 cell lines derived from human NSCLCs (A549, BEN, NCI-H226, NCI-H292, NCI-H460, NCI-H522, NCI-H596, NCI-H727, NCI-H838, NCI-H1299, NCI-H1975). Primary human normal bronchial epithelial cells (NHBE) were used as control of normal lung and set arbitrarily as 1. Expression analysis of miR-196a by quantitative RT-PCR showed that the levels of miR-196a were significantly increased in all but two NSCLC-derived cell lines compared to NHBE used as control normal cells.

Furthermore, we observed also that miR-196a is overexpressed in primary NSCLC samples [n=28; 12 squamous cell carcinomas (SCC), 8 carcinoids (CAR), 5 adenocarcinomas (ADC), 2 large cell carcinomas (LCC) and 1 adeno-squamous carcinoma (ADS)]. As controls, 6 matched normal samples were analyzed and set arbitrarily as 1. As shown in Figure 3C, the average levels of miR-196a were considerably higher in NSCLCs than in normal lung tissues (on average 14-fold).

### Relationship between activity of the PI3K/AKT pathway and the expression of miR-196a

The overexpression of miR-196a in NSCLC is a common event, and it is not extremely novel per se. On the contrary, the novelty of this study resides in positioning miR-196a downstream the PI3K/AKT pathway. To further investigate the relationship between the PI3K/AKT pathway and miR-196a expression, first we correlated the levels of miR-196a with the activation status of the PI3K/AKT pathway in 7 NSCLC cell lines, of which the activation status of the PI3K/AKT pathway was known [12]. In this case, we found that 2 out of 3 pAKT negative cell lines presented low levels of miR-196a whereas 4/4 pAKT positive cell lines presented high levels of miR-196a. See Table 2.

**Table 2:** Correlation between pAKT and miR-196a expression in NSCLC cell lines

Lung cancer cell lines	p-AKT	miR-196a FC
**A549**	+	5.98
**BEN**	-	0.11
**H226**	-	0.46
**H292**	+	14.31
**H460**	+	14.43
**H522**	-	57.60
**H596**	+	2.46

However, this correlation was not confirmed when we used *in silico* data downloaded from the public repository of The Cancer Genome Atlas (TCGA) consortium (http://cancergenome.nih.gov/). In fact, we exploited the TCGA dataset to correlate the presence of activating mutations in genes within the PI3K/AKT pathway with the expression of miR-196a in multiple NSCLCs. Samples were divided into 2 different groups: i) tumors mutated for AKT1, PIK3CA and PTEN (n=32), and ii) the remaining wild type tumors (n=391). We found that in the group of mutant samples, 19/32 (59.3%) showed high levels of miR-196a whereas in the group of wild type samples, 192/391 (49.1%) showed high levels of miR-196a. Therefore, no statistically significant correlation was observed by Fisher's Test.

Subsequently, we added to the group under analysis tumors harbouring KRAS mutations, an alteration known to activate PI3K/AKT signaling. In this case mutant samples became 158. Of these 81 showed high levels of miR-196a expression (51.2%) whereas in the remaining 265 non-mutant samples miR-196a was overexpressed in 130 (49%). Again Fisher's Test yielded non-significant values.

These contradictory results could be explained by the consideration that a correlation between mutated members of the PI3K/AKT pathway and miR-196a expression can be observed when simple systems such as cell lines are analyzed, but not when more complex and heterogeneous samples as tumors are considered.

A different approach to correlate the expression of miR-196a with the activity of the PI3K/AKT pathway was to determine the effects of suppressing the expression and/or activity of AKT isoforms in 2 different NSCLC cell lines (NCI-H460, A549) on miR-196a levels. To this aim we suppressed AKT1 and AKT2 expression in an established lung cancer cell line (NCI-H460) that harbours an activating mutation of PIK3CA (E545K) as reported [48]. In the work described in that manuscript, NCI-H460 cells were transduced with lentivirus expressing three different shRNAs to AKT1 and AKT2.

**Figure 4:**
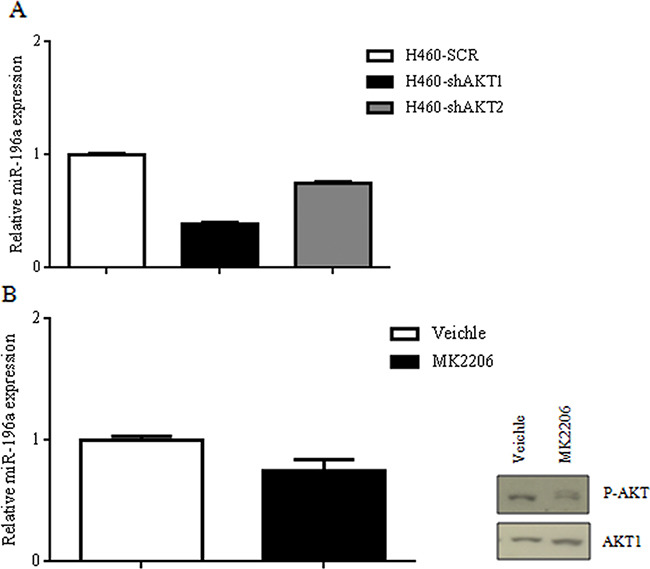
Suppression of AKT signaling on miR-196a expression in NSCLC cells **A**. Relative mRNA expression of miR-196a by qRT-PCR in H460SCR, H460-shAKT1 and H460-shAKT2 cells. **B**. Relative mRNA expression of miR-196a by qRT-PCR in A549 cells treated with MK2206 and immunoblot for the expression of p-AKT and AKT1.

Silencing of AKT1 in NCI-H460, as assessed by immunoblot [48], resulted in a marked reduction of miR-196a levels as detected by quantitative RT-PCR (Figure 4A), indicating that AKT kinases play a significant role in the expression of miR-196a in NSCLC cells. Similarly to AKT1 suppression, also the silencing of AKT2 in NCI-H460 cells produced a marked decrease in the levels of miR-196a as detected by quantitative RT-PCR (Figure 4A).

In addition, exposure of A549 NSCLC cells to MK2206, a pharmacological inhibitor of all AKT isoforms, markedly decreased AKT phosphorylation and significantly impaired miR-196a expression (Figure 4B). These results suggested that all the AKT isoforms expressed in the lung are able to increase miR-196a levels in NSCLC cells.

Finally, to investigate the presence of a mechanistic link between miR-196a and the PI3K/AKT pathway, we checked the promoter region of miR-196a for Transcriptional Factors’ binding sites, in particular those activated upon PI3K/AKT signaling. We reasoned that if the expression of miR-196a was under the transcriptional control of PI3K/AKT pathway, binding sites for Transcription Factors regulated by PI3K signaling in the promoter region of miR-196a should be identified.

Therefore, we performed a thorough analysis of statistically overrepresented MOTIF elements by Pscan (http://159.149.160.51/pscan/)[PSCAN], using RefSeq ID: NR_029582 as miR-196a identifier and *Homo sapiens* as source organism. The region analyzed was from −1000 to +0 bp with regard to the annotated transcriptional start site of the miR-196a promoter, and employing Jaspar 2016 as the database for the analysis. As a result, the output of the analysis returned a list of 50 Transcription Factors. See Supplementary Table S8. An element was considered to be significantly overrepresented if the P value was less than 0.05.

Among others, we found that the -1000/+0 promoter region of miR-196a contained the binding sites for FOXOq1, CREB3 and E2F, which are known to be activated by PI3K/AKT signaling [55–57] and thus are candidates to transcriptionally mediate PI3K/AKT-dependent regulation of miR-196a expression in NSCLC cells.

Overall these experiments indicate the existence of a tight loop between PI3K/AKT signaling and the expression of miR-196a in NSCLC cells that can be mediated by the binding of transcription factors regulated by PI3K/AKT to DNA sequences within the miR-196a promoter region.

### MiR-196a stimulates anchorage-dependent and -independent proliferation in NSCLC cells

Given the importance of AKT signaling in the pro-migratory phenotype of NSCLC cells, [58, 59] we investigated the role of miR-196a on anchorage-dependent and independent growth, migration and tumorigenic potential of human NSCLC cells. To this aim, we made use of human bronchial epithelial cells (BEAS-2B) and the corresponding derivative that express activated AKT1-E17K (BEAS-AKT1-E17K). See Supplementary Figure S3 for expression of miR-196a in BEAS-2B cells transduced with control (EV) or miR-196a expressing lentiviruses (Supplementary Figure S3A) and BEAS-AKT1-E17K cells transduced with control (EV) or antimiR-196a expressing lentiviruses (Supplementary Figure S3B).

**Figure 5:**
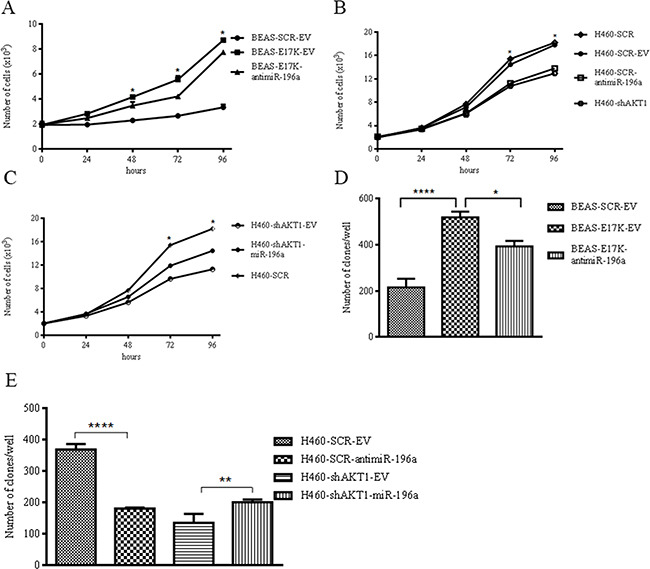
MiR-196a regulates anchorage-dependent and –independent growth of NSCLC cells **A**. MTT cell proliferation assay of BEAS-2B, BEAS-2B cells expressing antimiR-196a. Data are means ± SD calculated using triplicate samples in three separate experiments. p< 0.05. **B**. MTT cell proliferation assay of NCI-H460 cells expressing antimiR-196a. Data are means ± SD calculated using triplicate samples in three separate experiments. p< 0.05. **C**. MTT cell proliferation assay of NCI-H460-shAKT1 cells expressing miR-196a. Data are means ± SD calculated using triplicate samples in three separate experiments. p< 0.05. **D**. Soft agar colony formation assay of BEAS-SCR-EV, BEAS-E17K-EV, BEAS-E17K-antimiR-196a. Bars represent the means of triplicate experiments ± SD. p<0.01. As controls we used: BEAS cells that were infected with a lentivirus expressing a MISSION pLKO.1-puro Non-Target shRNA Control Plasmid DNA that contains an shRNA insert that does not target any known gene from any species (BEAS-SCR cells); BEAS-2B and derivatives cells were infected with a control lentivirus carrying an empty pre-microRNA Expression Construct Lenti-miR (EV) (BEAS-SCR-EV and BEAS-E17K-EV, respectively). **E**. Soft agar colony formation assay of NCI-H460, NCI-H460-antimiR-196a, NCI-H460-shAKT1 and NCI-H460-shAKT1-miR-196a. Bars represent the means of triplicate experiments ± SD. p<0.01. As controls we used: NCI-H460 cells that were infected with a lentivirus expressing a MISSION pLKO.1-puro Non-Target shRNA Control Plasmid DNA that contains an shRNA insert that does not target any known gene from any species (NCI-H460 SCR cells); NCI-H460 and derivatives cells were infected with a control lentivirus carrying an empty pre-microRNA Expression Construct Lenti-miR (EV) (NCI-H460-SCR-EV and NCI-H460-shAKT1-EV cells, respectively).

As an alternative approach, we suppressed miR-196a in the NSCLC cell line NCI-H460, which harbors a heterozygous activating mutation (E545K) in PIK3CA, resulting in activation of the PI3K/AKT pathway. See Supplementary Figure S3 for expression of miR-196a in NCI-H460 cells transduced with control (EV) or antimiR-196a expressing lentiviruses (Supplementary Figure S3C) and NCI-H460-shAKT1 cells transduced with control (EV) or miR-196a expressing lentiviruses (Supplementary Figure S3D). Here we demonstrate that suppression of miR-196a expression by use of antimiR-196a in BEAS-2B and NCI-H460 cells reduced *in vitro* anchorage-dependent and -independent growth, *in vitro* migration and *in vivo* tumor growth of cells subcutaneously injected into immunodeficient mice (n = 4/group). Analysis of cell proliferation showed that antimiR-196a transduced into BEAS-2B cells reduced the proliferative capability of BEAS-E17K cells. Indeed, the growth curves in Figure 5A indicated that after 96 hours the average number of BEAS-SCR-EV cells was 3.3±0.22×10^3^, the average number of BEAS-E17K-EV cells was 8.7×10^3^±1.8×10^2^ and the average number of BEAS-E17K-antimiR-196a cells was 7.7±0.19×10^3^.

In NCI-H460 cells, antimiR-196a transduction significantly decreased cell proliferative rate with effects similar to, or even more pronounced of, those exerted by interference of AKT1 in NCI-H460 cells (NCI-H460-shAKT1). The growth curve in Figure 5B showed that after 96 hours the average number of control NCI-H460 cells was 17.8±0.2×10^3^, the average number of NCI-H460-shAKT1 cells was 12.9±0.4×10^3^ and the average number of NCI-H460-SCR-antimiR-196a cells was 13.8±0.4×10^3^. Conversely, overexpression of miR-196a in NCI-H460sh-AKT1 cells caused a partial rescue of growth rate inhibited by AKT1 interference (Figure 5C).

In addition, miR-196a promoted anchorage-independent growth *in vitro*, as BEAS-SCR-EV cells generated 216±36.8 colonies/well, BEAS-E17K-EV generated 519±46.5 colonies/well and BEAS-E17K-antimiR-196a generated 393±24 colonies/well (Figure 5D; see Supplementary Figure S4 for representative images). Expression of miR-196a promoted anchorage-independent growth *in vitro* also in BEAS-2B cells, whereas its inhibition obtained by transducing a lentivirus expressing an antimiR-196a, partially counteracted the pro-mitogenic effects induced by mutant AKT1 in BEAS-AKT1-E17K cells (Figure 5D).

In NCI-H460 cells inhibition of miR-196a by use of the specific antimiRNA markedly reduced the number of colonies/well (from 368±17.8 to 180±3.7). On the other hand, this miRNA increased the number of colonies/well when expressed into NCI-H460-shAKT1 cells (200±9.1 vs. 134.5±28.9; Figure 5E; see Supplementary Figure S4 for representative images).

These results indicate that miR-196a is able to promote anchorage-dependent and -independent proliferation of NSCLC cells.

### MiR-196a stimulates migration and tumorigenicity in NSCLC cells

**Figure 6:**
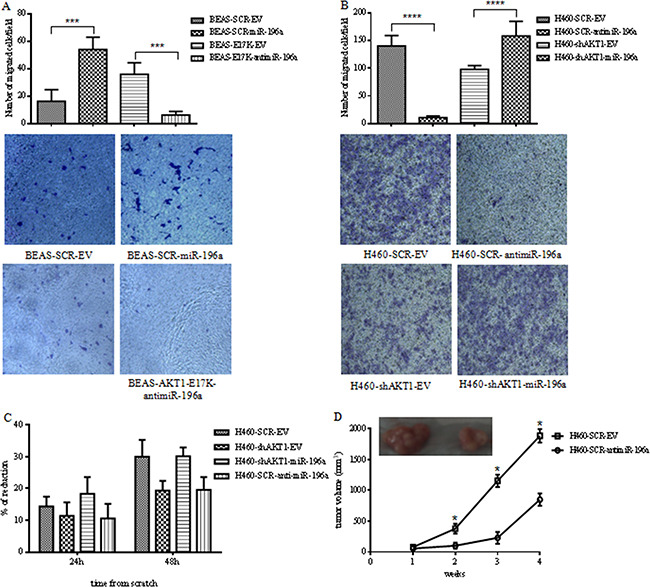
MiR-196a regulates migration and tumorigenicity of NSCLC cells **A**. Boyden chamber migration assay of control BEAS-SCR-EV, BEAS-SCR-miR-196a, BEAS-E17K-EV and BEAS-E17K-antimiR-196a cells. The graph represents the mean number (± SD) of migrated cells/field. Representative images of migrated BEAS-2B and derivative cells. Magnification 10X; p<0.001. **B**. Boyden chamber migration assay of control NCI-H460-SCR-EV, NCI-H460-shAKT1-EV, NCI-H460-SCR-antimiR-196a and NCI-H460-shAKT1-miR-196a cells. The graph represents the mean number (± SD) of migrated cells/field. Representative images of migrated NCI-H460 and derivative cells. Magnification 10X; p<0.001. **C**. Wound healing assay of NCI-H460-SCR-EV, NCI-H460-shAKT1-EV, NCI-H460-shAKT1-miR-196a and NCI-H460-SCR-antimiR-196a cells. The graph represents the percentage of wound area reduction after 24h and 48h from scratch. **D**. Tumor growth of NCI-H460-SCR-EV and NCI-H460-antimiR-196a cells injected into nude mice (n=4/group); data are shown as mean ± SD.

We then investigated whether miR-196a regulated migration in NSCLC cells and mediated, at least in part, the pro-migratory effects of activated AKT by Boyden chamber assays and wound assays. First, we found that expression of miR-196a increased migration of BEAS-2B cells of approximately 3-fold, whereas its inhibition by the antimiRNA in BEAS-AKT1-E17K cells counteracted the pro-migratory effects induced by mutant AKT1 (Figure 6A). On the other hand, inhibition of miR-196a markedly reduced NCI-H460 cell migration, assessed in Boyden chamber assays (140±18.9 *vs* 10.5±0.3 migrated cells; Figure 6B), whereas adoptive expression of miR-196a in NCI-H460-shAKT1 cells rescued the impairment of cell migration induced by interference of AKT1 (158±26.8 *vs* 98±6.7).

These results were confirmed by wound assay of confluent monolayers of control NCI-H460, NCI-H460 transduced with a lentivirus expressing antimiR-196a, NCI-H460-shAKT1, NCI-H460-shAKT1 transduced with miR-196a. As shown in Figure 6C, miR-196a suppression caused an almost 2-fold reduction in the capability of NCI-H460 cells to migrate at 48h after wounding. In agreement with these results, we found that miR-196a rescued the reduction of migrated cells induced by suppression of AKT1 in NCI-H460-shAKT1 cells. See Supplementary Figure S5 for representative images of wound closure.

Finally, suppression of miR-196a by use of antimiR-196a markedly reduced *in vivo* tumor expansion of NSCLC cells injected into immunodeficient mice (n=4/group) (Figure 6D), indicating that miR-196a plays a significant role in the malignant behavior of NSCLC cells.

### Pro-tumorigenic activities exerted by miR-196a in NSCLC cells are partly mediated by FoxO1, p27 and HOXA9

To further dissect the biological contribution of miRNAs to aberrant PI3K/AKT signaling in NSCLC cells, we searched for miR-196a targets using TargetScan, microRNA.org and miRANDA. Target RNAs were selected on the basis of the number of databases that identified them. Using these criteria, and in consideration of previously published work [52, 60–62], we selected the transcription factor FoxO1 and cyclin-dependent kinase inhibitor p27 as effectors of miR-196a in NSCLC cells. This was strengthened also by our findings that this miR-196a stimulates proliferation in NSCLC cells (see above).

**Figure 7:**
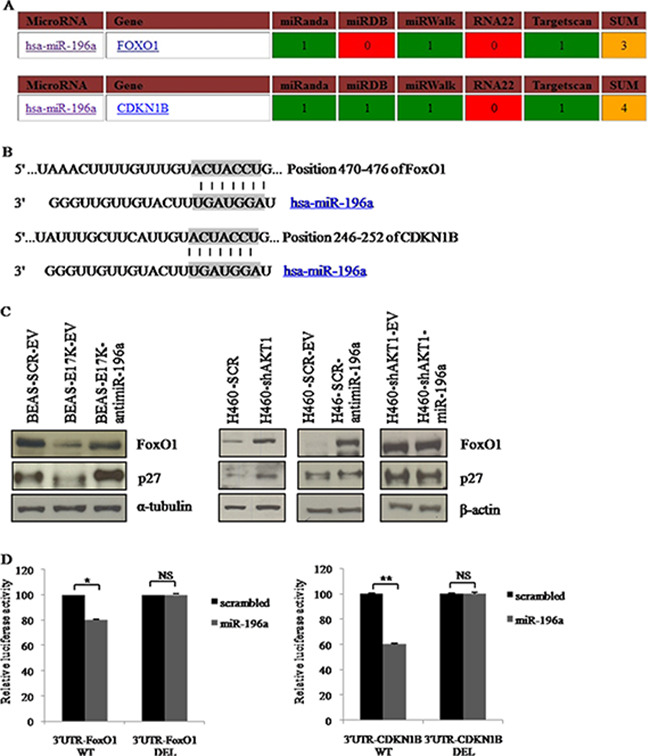
Role of predicted target genes of miR-196a in NSCLC cells **A**. Target prediction of miR-196a. **B**. Base-pairing between the seed sequence of miR-196a and the 3′UTR of FoxO1 and p27. **C**. Immunoblot analysis of FoxO1 and p27 as indicated. **D**. Left panel, Luciferase assay of plasmids 3′UTR-FoxO1-WT and 3′UTR-FoxO1-DEL transfected into HEK-293 cells with pre-miR-196a or a no-targeting scrambled (SCR) oligonucleotides; right panel, Luciferase assay of plasmids 3′UTR-CDKN1B-WT and 3′UTR-CDKN1B-DEL transfected into HEK-293 cells with pre-miR-196a oligonucleotide or a no-targeting scrambled oligonucleotide (SCR).

In Figure 7A are indicated the bioinformatic databases that support target prediction. Figure 7B shows the base-pairing of the seed sequence of miR-196a in the 3′UTR of FoxO1 and p27 encoding genes. We confirmed that FoxO1 and p27 were *bona fide* targets of miR-196a in NSCLC cells, by performing immunoblot analyses in BEAS-2B cells expressing miR-196a and in NCI-H460 cells in which the endogenous expression of miR-196a had been suppressed by use of anti-miR-196a (Figure 7C). Notably, BEAS-2B cells expressing AKT1-E17K showed similar modulation of p27 and FoxO1 proteins as BEAS-2B cells expressing miR-196a, and NCI-H460 cells interfered for AKT1 showed similar modulation of CDKN1B and FoxO1 proteins as H460 cells-antimiR-196a.

Finally, we searched for additional experimental evidence that FoxO1 and p27 mRNAs were targets of miR-196a by performing Luciferase assay. To this aim, HEK-293 cells were co-transfected with a pre-miR-196a oligonucleotide or a scrambled oligonucleotide (SCR), together with the 3′-UTR of FoxO1 (Luc-3′UTRFoxO1) or of p27 (Luc-3′UTR-CDKN1B) fused to Luciferase (Figure 7D, left and right, respectively). We found that the transfection of pre-miR-196a oligonucleotide, but not of the scrambled oligonucleotide, reduced the luciferase activity of approximately 20% for FoxO1 and 40% for p27.

To confirm that binding to seeds in the 3′UTR of FoxO1 and p27 was critical for miR-196a activity, we deleted the seed sequences of miR-196a in both target genes (3′UTR-FoxO1-DEL and 3′UTR-CDKN1B-DEL) and performed Luciferase activity assay. These experiments demonstrated that the deletion of the seeds in both FoxO1 and p27 suppressed the activity of miR-196a, thus suggesting that miR-196a reduces the expression of FoxO1 and of p27 by directly binding the seed sequences in their 3′-UTR (Figure 7D, left and right, respectively).

Subsequently, we investigated the relationship between miR-196a and its targets FoxO1 and p27.

**Figure 8:**
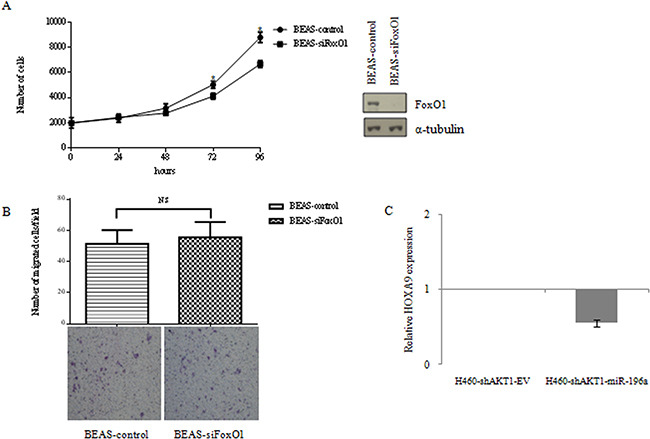
**A**. MTT cell proliferation assay of BEAS-2B cells silenced for FoxO1 and immunoblot for FoxO1 expression. Data are means ± SD calculated using triplicate samples in three separate experiments. p< 0.05. **B**. Boyden chamber migration assay of control BEAS-2B cells silenced for FoxO1. The graph represents the mean number (± SD) of migrated cells/field. Representative images of migrated BEAS-2B cells silenced for FoxO1. Magnification 10X. **C**. Relative HOXA9 expression by qRT-PCR in NCI-H460-shAKT1-EV, NCI-H460-shAKT1-miR-196a cells.

As to FoxO1, we have silenced FoxO1 with siRNA. Analysis of cell proliferation by MTT and of cell migration through Boyden chamber assay, demonstrated that FoxO1 expression has a moderate effect on cell proliferation of NSCLC cells and has no effect at all on migration (Figure 8A–8B).

As to p27, we have previously demonstrated that its expression in lung epithelial cells inhibits completely growth and, on the contrary, the suppression of its expression by shRNA in BEAS-AKT1-E17K significantly increased cell proliferation and reduced cell migration [47]. These results indicate that both p27 and FoxO1 regulate proliferation in BEAS-2B cells and that p27, but not FoxO1, is involved in the regulation of migration.

Another candidate for the regulation of migration in NSCLC cells is HOXA9 [63]. Data from the literature indicate that HOXA9, a negative regulator of migration in NSCLC cells, is a direct target for miR-196a [63, 64]. Thus, we investigated whether HOXA9 expression was regulated by miR-196a in NSCLC cells. Quantitative RT-PCR analysis indicated that adoptive expression of miR-196a in H460-shAKT1 cells induced a consistent down-regulation of HOXA9 expression compared to the empty vector (Figure 8C).

The finding that adoptive expression of miR-196a in NSCLC cells induced a consistent down-regulation of HOXA9, suggests that miR-196a-mediated HOXA9 down-regulation contributes to the pro-migratory effects exerted by miR-196a in NSCLC cells, though we can not formally exclude that HOXA9 regulation by miR-196 may take part also to the regulation of proliferative pathways.

## DISCUSSION

Changes in the expression of miRNAs are associated with many human diseases including cancer [65–68], which has suggested a potential diagnostic or prognostic utilization of miRNAs in cancer [69]. However, the molecular mechanisms whereby miRNA expression is deregulated in cancer remain to be elucidated in several cases. Accordingly, although aberrant PI3K signaling is a frequent event in NSCLC cell lines [14–16] and primary tumors [17–21], there is no information so far about the role of miRNAs in mediating the effects exerted by oncogenic signaling elicited by activated PI3K pathway.

In this study, we have identified a group of miRNAs whose expression in lung cancer cells is increased/decreased when the PI3K/AKT pathway is oncogenically activated. In addition, we demonstrated that one such miRNA, miR-196a, mediates some of the transforming activities of activated PI3K/AKT pathway in NSCLC cells. These findings are noteworthy, as they shed light on the role exerted by miRNAs in aberrant PI3K signaling in NSCLC and, at the same time, provide experimental evidence that miR-196a can stimulate anchorage-dependent and -independent proliferation and migration in NSCLC cells downstream PI3K, thus ameliorating the comprehension of the pathogenesis of this neoplasia.

The data presented in this study demonstrate that aberrant PI3K signaling - induced by the adoptive expression of active mutant alleles of AKT1 or PI3KCA or by PTEN silencing - deregulates expression of 24 miRNAs in human bronchial epithelial cells. Among these, the behavior of miR-16-1*/miR-16-5p and miR-203 was concordant with their putative anti-oncogenic properties, whereas regulation of miR-196a was concordant with a supposed oncogenic effect. In particular, miR-196a, the DEM that showed the highest average fold change (>2) in all three derivatives of BEAS-cells (AKT1-E17K, BEAS-PIK3CA-E545K and BEAS-shPTEN), emerges as a critical player of PI3K signaling in lung cancer, regulating several oncogenic molecular networks. Expression of miR-196a expression in immortalized human bronchial epithelial cells (BEAS-2B) and/or its silencing in NSCLC cells harboring a PIK3CA mutation (NCI-H460), affected anchorage-dependent and -independent proliferation, migration and tumor growth in xenografts, further extending previous work performed in SPC-A1 and/or A549 cells *in vitro* [54].

MiR-196 is transcribed from three different genes denoted miR-196a-1, miR-196a-2 and miR-196b, with its expression strictly controlled in normal tissues [70]. Conversely, dysregulation of miR-196 expression is frequently observed in many cancer types including pancreatic, breast, colorectal, esophageal and lung carcinomas, as well as leukemias [8, 11–13, 70]. The results presented in this study, indicate that miR-196a is significantly overexpressed in human NSCLC-derived cell lines (n=9/11) and primary lung cancer samples (n=19/28), in particular, it is overexpressed in 9/12 SCC, 4/8 CAR, 3/5 ADC, 2/2 LCC, 1/1 ADS. It should be noted that, while high miR-196a levels have been unequivocally associated with advanced clinical stage of disease and lymph-node metastasis, [71] the clinical significance of miR-196a up-regulation in NSCLC remains to be further confirmed, due to the small number of samples analyzed in this study.

The full spectrum of miR-196 actions in the different types of cancer cells is largely unknown. A complete understanding of the role that miR-196 might play in cancer requires the identification of its targets. MiRNAs regulate gene expression by inhibiting synthesis of specific proteins *via* base-pairing of their 5′ nucleotides with the 3′UTR of target mRNA [70]. Recent work has shown that miR-196 targets FoxO1 in cervical cancer cells [62] and p27 in gastric cancer cells [52]. Based on these evidences, and on the predicted binding sites for miR-196a identified in this study, we observed that FoxO1 and p27 are direct targets of miR-196a in NSCLC cells.

Taken together, these results indicate that miR-196 plays a critical role in the pathogenesis of lung cancer, and that at least some of the oncogenic activities of miR-196a may depend on suppression of the expression of the transcription factor FoxO1 and/or the cyclin-dependent kinase inhibitor p27. Importantly, miR-196a has multiple effects on p27 levels - both direct and indirect. In fact, besides being controlled by miR-196, p27 expression is also regulated by FoxO transcription factors [72–74].

**Figure 9:**
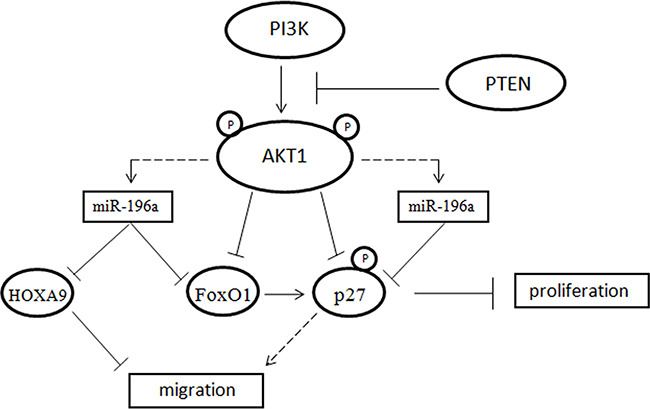
Model explaining the relationship among PI3K, AKT1, miR-196a, FoxO1, p27 and HOXA9

By suppressing FoxO1 and p27, miR-196a would thus stimulate cell cycle progression in NSCLC cells. As deregulation of CDK inhibitors such as p27 is commonly observed in tumor cells [75], overexpression of miR-196 may represent one mechanism whereby the activity of this important cell cycle inhibitor is impaired in cancer. Accordingly, we have previously demonstrated that suppression of p27 expression in lung epithelial cells completely inhibits growth and, on the contrary, the suppression of its expression by shRNA in BEAS-AKT1-E17K significantly increased cell proliferation and reduced cell migration [47]. On the other hand, silencing of FoxO1 had a moderate effect on cell proliferation and no effect at all on migration. Finally, the inhibition of HOXA9 by miR-196a may contribute to stimulate migration effects in NSCLC cells (Figure 9).

In conclusion, the data reported here provide strong support to the concept that expression of miR-196a is modulated by the most common alterations that contribute to the development of NSCLC, including activating mutations of PIK3CA (E545K) and AKT1 (E17K), and/or the loss of PTEN expression. MiR-196a could stimulate proliferation/migration by directly targeting FoxO1, p27 and HOXA9 and thus plays an important role in mediating the effects exerted by aberrant activation of PI3K/AKT signaling.

## MATERIALS AND METHODS

### Patients

Patient accrual was conducted according to internal Review Board of the INT Fondazione Pascale (Naples, Italy) (CEI 556/10 of 12/3/2010). The study was approved by the internal Review Board of the AOU Mater Domini/University Magna Graecia (Catanzaro, Italy) in the meeting of 16/3/2011. Written informed consent was obtained from all participants to the study.

Archive material from 28 patients diagnosed of NSCLC was obtained from INT “Fondazione Pascale” (Naples, Italy).

### Cell culture

A549, BEN, NCI-H226, NCI-H292, NCI-H460, NCI-H522, NCI-H596, NCI-H727, NCI-H838, NCI-H1299, NCI-H1975 human lung cancer cell lines were purchased from ATCC-LGC Promochem (South West London, UK) and maintained in RPMI 1640 (Lonza Walkersville Inc., MD) supplemented with 10% fetal bovine serum (Sigma-Aldrich, St.Luis, MO, USA) and 100 units/ml penicillin-streptomycin (Lonza Walkersville Inc.). A549 cells were treated with MK2206 (Selleckchem, Huston, TX) at a concentration of 1μM for 3 hours.

NHBE, Normal Human Bronchial Epithelial cell line, was purchased from ATCC-LGC Promochem (South West London) and maintained in BEGM medium (Lonza).

BEAS-2B, immortalised human bronchial epithelial cell line was purchased from Chambrex (Milan, Italy) and grown according to the manufacturer's protocol [76]. Transfection of siRNA was performed with MISSION^®^siRNA Transfection Reagent (S1452, Sigma Aldrich) by using 10nM of control (SIC007, Sigma Aldrich) and 10 nM of FoxO1 siRNA (PDSIRNA2D, Sigma Aldrich) according to the manufacturer's protocol.

### Generation of stably transfected cell lines

Human pre-microRNA Expression Construct Lenti-miR-196a-1 (PMIRH196a1PA-1) and human miRZip-196a anti-miR-196a microRNA Construct (MZIP196a-PA-1) (System Biosciences, Mountain View, CA, USA) were used to generate lentiviral particles in HEK 293T packaging cells. As control, Scramble control hairpin in pCDH-CMV-MCS-EF1-copGFP was used (PMIRH000PA-1, System Biosciences). HEK293T cells grown to 75% of confluency in culture (Corning Tewksbury, MA, USA) were transfected using the calcium phosphate precipitation method employing 13 μg of one of the two plasmids, 18 μg of pCMV-deltaR8.91 and 12 μg of pCMV-VSVG [77]. The efficiency of transfection was evaluated by the analysis of transfected cells with a fluorescence microscope, due to the presence of the EGFP gene in the constructs for micro-RNA overexpression or knockdown. At 24 h after transfection, 4.5 ml of DMEM containing 10% FBS were added to the cultures. For lentiviruses producted to infect BEAS-2B cells, DMEM was complemented with 3% FBS. Supernatants harvested 48 h and 72 h post-transfection were filtered (0.22 μM, Millipore, Billerica, MA, USA) and directly used for infection cycles.

H460 (1×10^5^ cells/well) and BEAS-2B cells (1.5×10^5^ cells/well) were plated on six-well plates (Corning) 24 h before infection. A measure of 2ml of lentivirus supernatant containing 8 mg/ml polybrene (Sigma-Aldrich) were added to the cells. Spinoculation was performed at 2000 rpm, 30°C for 45 min. Two rounds of 12h infection were carried out. At the end of the infection procedure, supernatant containing lentiviruses was replaced with complete culture media. Fluorescent clones were picked up and amplified.

### RNA extraction

Total RNA isolation from cells and clinical samples was performed with Trizol Reagent (Invitrogen, Carlsbad, CA, USA) according to the manufacturer's protocol. RNA integrity was assessed by denaturing agarose gel electrophoresis.

### miRNA Bead Chip microarray

RNA concentration was determined with a Nanodrop (NanoDrop, Wilmington, Delaware, USA) spectrophotometer. From each sample, technical replicates were produced and 600ng RNA were hybridized for 18hrs to Human v2 MicroRNA Expression BeadChips (Illumina Inc., San Diego, CA, USA)as described earlier [78]. BeadChips were dried and scanned with an Illumina Bead Array Reader (Illumina Inc.). For data analysis, the intensity files were loaded into the Illumina Genome Studio V2011.1 software for quality control and miRNA expression analysis. First, the quantile normalization was performed using GeneSpring 13.1.1 (Agilent Technologies) to correct systematic errors. For differential expression analysis, technical replicates of each sample were grouped together to identify differentially expressed miRNAs having a fold change ≥ 1.5 and P < 0.05, as determined by Welch t test statistical analysis.

### Gene characterization, enriched pathways and bibliographic networks discovery

The DEMs list was used to evaluate the functional behavior in terms of Biological Processes and Molecular Function, Development Function and Disease and Disorder terms. The degree of enrichment was statistically evaluated to determine whether an observed level of annotation for a group of genes is significant. In particular, for each term, a q-value was computed by the Hypergeometric test (p≤0.05) and corrected using False Discovery Rate (FDR). The terms with a q-value exceeding the significance threshold were then selected as representative. Pathway and network analysis were performed using Ingenuity Pathway Analysis (IPA, Ingenuity Systems). The dataset was mined for significant pathways with the IPA library of canonical pathways, and networks were generated by using IPA as graphical representation of the molecular relationships between genes and gene products. The significance of the association between the list of DEMs and the Canonical Pathway was measured using a Fisher's exact test to calculate a p-value (p≤0.05). Fisher's exact test results were also corrected for multiple testing using FDR. In networks miRNA, genes or gene products are represented as nodes, and the biological relationship between two nodes is represented as an edge (line). All edges are supported by at least one reference from the literature, from a textbook, or from canonical information stored in the IPA Knowledge Base. Human, mouse, and rat orthologs of a gene are stored as separate objects, but are represented as a single node in the network. The network building's algorithm determines a statistical score for each network. This is done by comparing the number of focus genes that contribute to a given network relative to the total number of occurrences of those genes in all networks or pathways stored in the IPA Knowledge Base. The intensity of color in the networks indicates the degree of down-regulation (green) or up-regulation (red) of expression. Nodes are displayed using various shapes that represent the functional class of gene products.

### Analysis of miR-196a promoter

Analysis of statistically over-represented MOTIF elements was conducted by Pscan (http://159.149.160.51/pscan/) [PSCAN]. Significance of the results were assessed by calculatingp-values computed by Pscan with a z-test, which associates the probability of obtaining the same score on a random sequence set [PSCAN] with each profile. An element was considered to be significantly over-represented if the p-value was less than 0.05.

### Quantitative real time RT-PCR (qRT-PCR)

Total RNA was reverse transcribed to cDNA using the Universal cDNA Synthesis kit II (Exiqon, Denmark). The q-PCR reaction was performed using the ExiLENT SYBR® Green Master Mix kit (Exiqon) utilized withLNA™ primers (Exiqon). The amount of each miRNA was normalized to the U6 RNA using the equation 2^−ΔCT^, where ΔCT = (CTmiRNA- CTU6RNA).

### Plasmids

For luciferase assays, the regions of 3′UTR of FoxO1 and CDKN1B containing the miRNA binding sites were cloned in pGL3 control vector (Promega, Milan, Italy) at XbaI restriction site, downstream luc gene. The 3′UTR regions of target genes were amplified from human blood cells genome by PCR reaction using the following primers:

primer forward FoxO1: CCCAATGTGTGCAGGT TATG; primer reverse FoxO1: AGGTCCAAGGCTGT TCAATG; primer forward CDKN1B: TTCATGGAATGG ACATCCTGT; primer reverse CDKN1B: CCTTCCCCAA AATTGCTTCT.

After cloning in DH5α bacteria strain, positive clones were identified by PCR on colonies using the same forward oligonucleotides described before and a reverse oligonucleotide that aligns on pGL3 control vector. Clones that were positive for PCR were screened also for digestion with XbaI. Only clones that were positive for both screenings were sequenced. Deletion of the seed sequence was performed by PCR.

These plasmids were used to transfect HEK293 cells with siPORT (Ambion Life Technologies, Paisley, UK) in the presence of mature microRNA-196a to evaluate the true bind of this miRNA to its own site on 3′UTR region of predicted target genes. Luciferase activity was assayed with a dual luciferase assay system (Promega, Milan, Italy) as described in the manufacturer instructions.

### Western blot and antibodies

Whole cell protein extracts were prepared by homogenizing cells in NP-40 lysis buffer (10 mMTris–HCl (pH 7.5), 150 mMNaCl, 1% NP-40) containing protease inhibitors. Lysates were cleared by centrifugation and proteins were separated by SDS-PAGE. Proteins from gels were transferred to nitrocellulose membranes with TurboBlot System (Bio-rad Laboratories, Hercules, CA). The membranes were blocked with 5% nonfat dry milk in 0.05% Tween-20 in TBS (TBST) for 1h at room temperature and then incubated with primary antibodies overnight at 4°C. After washing with TBST for three times (5 minutes each), the membranes were incubated for 1h at room temperature with 1:2500 diluted peroxidase-conjugated anti-rabbit IgG or peroxidase-conjugated anti-mouse IgG (GE Healthcare, Milan, Italy) as a secondary antibody. For detection, enhanced chemiluminescent reaction (GE Healthcare) was performed according to the manufacturer's instructions.

Anti-phospho-AKT (Ser473) (#4058), anti-AKT1 (#2938), anti-FoxO1 (#2880), anti-PI3K-p110 (#4255), were purchased from Cell Signaling Technology (Denver, MA, USA); anti-p27 antibody (C19) was purchased from Santa Cruz Biotechnology (Santa Cruz, CA, USA); anti-β-actin (#A2228) was purchased from Sigma-Aldrich; anti-α-tubulin (Ab-2 DM1A, #MS-581-P0).

### *In vitro* proliferation assay

Cell proliferation was assayed by MTT [3-(4,5-dimethylthiazol-2-yl)-2,5-diphenyltetrazoliumbromide; Sigma-Aldrich] reduction. Cells were plated in 96-well flat-bottomed microtiter plates (200 μl cell suspensions, 2×103/well) and incubated with MTT substrate (5 mg/ml) for 2 h. Every 24 hours, the culture medium was removed and anhydrous 2-propanol was added. The optical density was measured at 570 nm.

### Wound healing assay

For wound healing assay cells were plated at equal density in six-well plates in duplicate and grown to confluence. Wounds were then generated with a sterile pipette, cells were rinsed twice with PBS, and fresh culture medium was added. Areas of wound were marked and photographed at 0 and 48h (Nikon Eclipse TE 2000-S). Wound area was measured with ImageJ software. Five different wound areas/well were analyzed.

### Migration assay

Transwell migration assay was performed using six-well Transwell polycarbonate filters (Sigma-Aldrich) with 8-μm pore size. Cells (5×10^4^) were seeded in the upper chamber of the Transwell insert and incubated for 48h at 37°C in serum-free medium. Cells that did not migrate through the pores were manually removed with a cotton swab. Cells that migrated to the bottom of the membrane were fixed in cold methanol for 10 min and then stained with 0.01% crystal violet in 20% ethanol. Migrated cells were counted in 5 fields/well in triplicate experiments.

### Soft agar assay

Cells (2.5×10^3^ NCI-H460 cells; 5×10^3^ BEAS-2B cells) were suspended in RPMI or BEBM, containing 0.35% low-melting agarose (Type VII, Sigma-Aldrich) and seeded onto 0.5% low-melting agarose in six-well tissue culture plates. Colonies were scored after 3 weeks by staining with 0.1% crystal violet (Sigma-Aldrich) in methanol in 5 randomly selected fields/well, at 4X magnification. Quantification and SD were obtained from results of three independent experiments.

### *In vivo* tumorigenic assay

NCI-H460 (2.5×10^6^) were suspended in 50μl of PBS and 50μl of Matrigel Low Growth Factor (BD Biosciences, San Jose, CA, USA) and subcutaneously injected into the flank of 6-week-old nude mice (Charles River, West Germany) in quadruplicates. Every 7 days tumor size was measured with a caliper.

### Statistical analysis

Data presented are the means ± SD of n independent assays or replicates as indicated in the text. Continuous variables were analyzed by Student's t-test or ANOVA test. Significance was calculated by Prism 5 Software (San Diego, CA, USA). Significance was set at p ≤ 0.05 (*), p ≤ 0.01 (**), p ≤ 0.001 (***) and p ≤ 0.0001 (****), respectively.

## SUPPLEMENTARY MATERIALS FIGURES AND TABLES


















