# Correction: Analysis of miRNA profiles identified miR-196a as a crucial mediator of aberrant PI3K/AKT signaling in lung cancer cells

**DOI:** 10.18632/oncotarget.28237

**Published:** 2022-05-25

**Authors:** Ilaria Guerriero, Daniela D’Angelo, Pierlorenzo Pallante, Mafalda Santos, Marianna Scrima, Donatella Malanga, Carmela De Marco, Maria Ravo, Alessandro Weisz, Carmelo Laudanna, Michele Ceccarelli, Geppino Falco, Antonia Rizzuto, Giuseppe Viglietto

**Affiliations:** ^1^Biogems.c.ar.l., Istituto di Ricerche Genetiche “Gaetano Salvatore”, Ariano Irpino, Avellino, Italy; ^2^Istituto per l’Endocrinologia e l’Oncologia Sperimentale, IEOS-CNR, c/o Dipartimento di Medicina Molecolare e Biotecnologie Mediche, Università “Federico II” of Napoli, Napoli, Italy; ^3^Dipartimento di Medicina Sperimentale e Clinica, Università Magna Graecia of Catanzaro, Italy; ^4^Laboratorio di Medicina Molecolare e Genomica, Dipartimentodi Medicina e Chirurgia, Università di Salerno, Baronissi, Italy; ^5^Department of Biological and Environmental Studies, Università del Sannio, Benevento, Italy; ^6^Dipartimento di Biologia, Università degli Studi di Napoli Federico II, Complesso Universitario Monte S.Angelo, Napoli, Italy; ^7^Dipartimento di Scienze Mediche e Chirurgiche, Università Magna Graecia of Catanzaro, Italy

**This article has been corrected:** In Figure 6B, the H460-SCR-EV image (upper left panel) contains accidental overlaps from the H460-shAKT1-miR-196a image (lower right panel). The corrected Figure 6B, produced using the original data, is shown below. The authors declare that these corrections do not change the results or conclusions of this paper.

Original article: Oncotarget. 2017; 8:19172–19191. 19172-19191. https://doi.org/10.18632/oncotarget.13432

**Figure 6:**
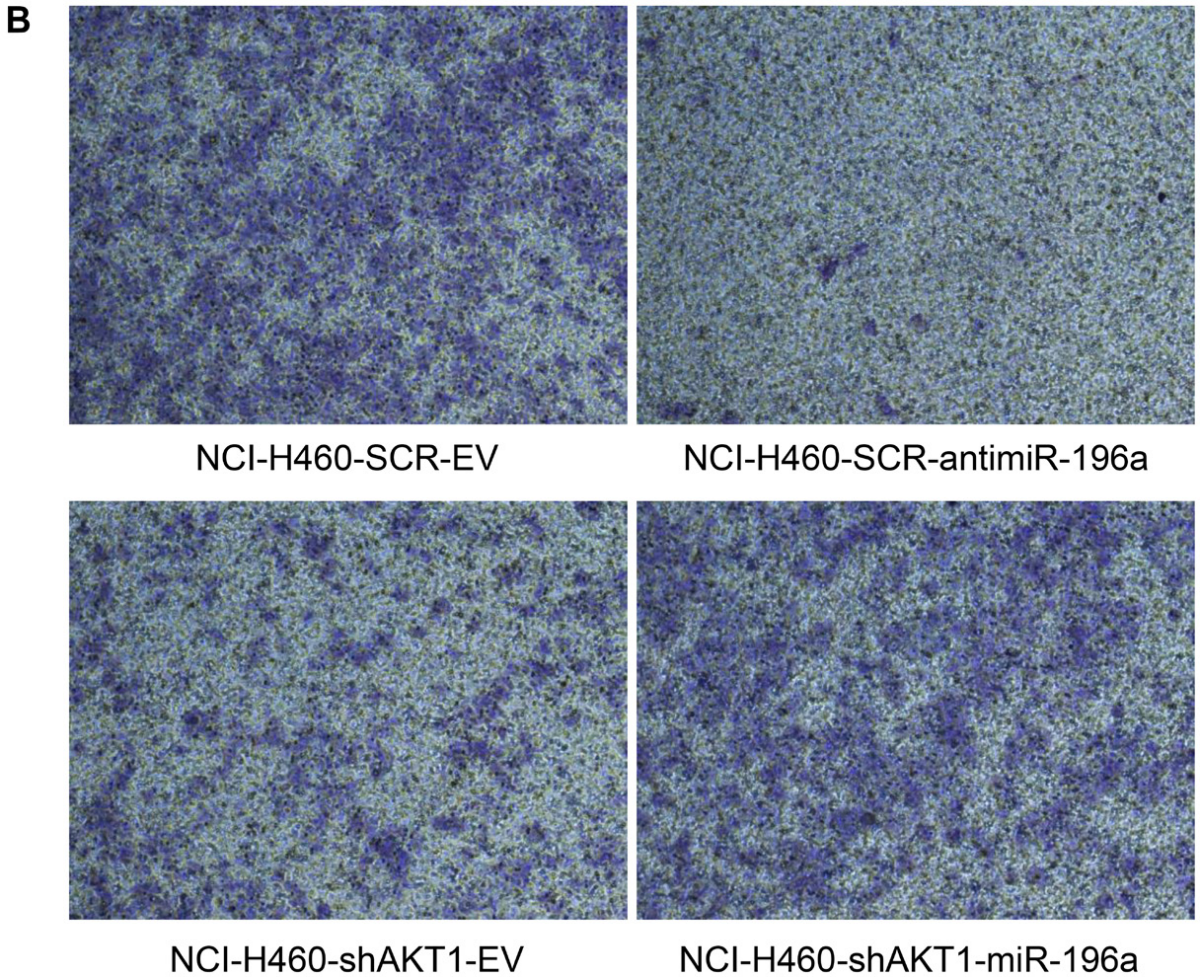
MiR-196a regulates migration and tumorigenicity of NSCLC cells. (**B**) Boyden chamber migration assay of control NCI-H460-SCR-EV, NCI-H460-shAKT1-EV, NCI-H460-SCR-antimiR-196a and NCI-H460-shAKT1-miR-196a cells. The graph represents the mean number (±SD) of migrated cells/field. Representative images of migrated NCI-H460 and derivative cells. Magnification 10×; *p* < 0.001.

