# Supplementary material for: Analysis of miRNA profiles identified miR-196a as a crucial mediator of aberrant PI3K/AKT signaling in lung cancer cells
Source: Oncotarget. 2016 Nov 17;8(12):19172–91. doi: 10.18632/oncotarget.13432 (PMC5386676; doi:10.18632/oncotarget.13432)
Supplement: Supplementary file 1 [file oncotarget-08-19172-s001.pdf]

Analysis of miRNA profiles identified miR-196a as a crucial mediator of aberrant PI3K/AKT signaling in lung cancer cells

SUPPLEMENTARY FIGURES AND TABLES

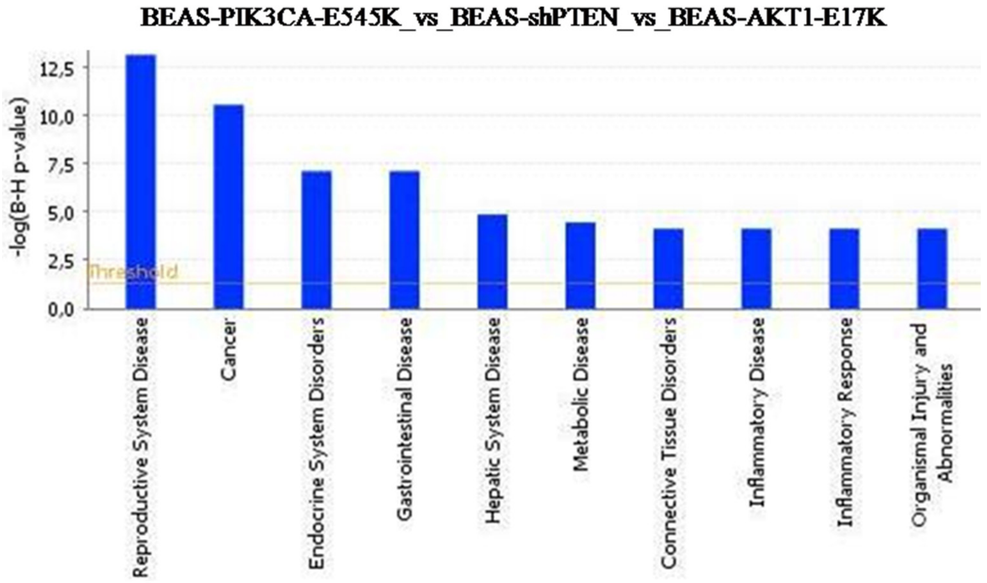

Supplementary Figure S1: Top Bio-functions identified by IPA in the DEMs common to BEAS-PIK3CA-E545K, BEAS-AKT1-E17K and BEAS-shPTEN.

BEAS-PIK3CA-E545K_vs_BEAS-shPTEN_vs_BEAS-AKT1-E17K-Network 1

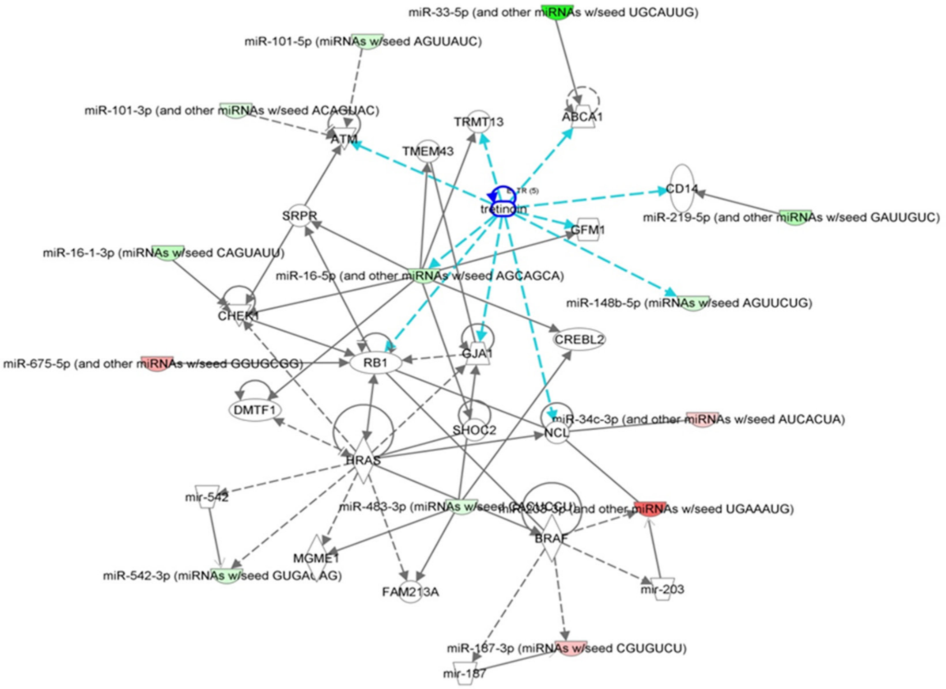

BEAS-PIK3CA-E545K_vs_BEAS-shPTEN_vs_BEAS-AKT1-E17K-Network 2

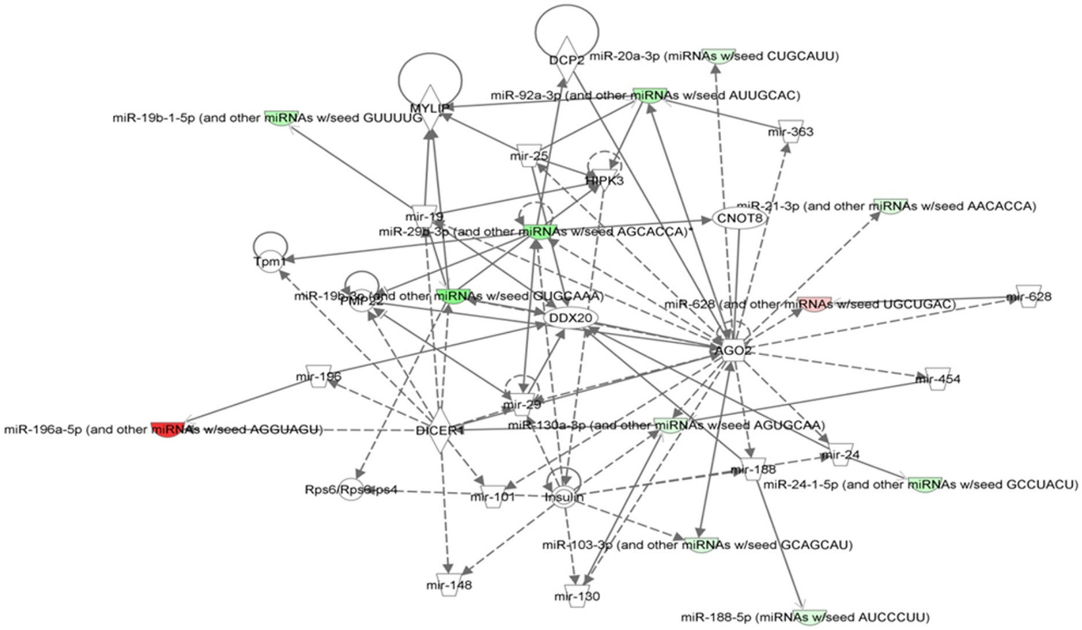

Supplementary Figure S2: Network analysis was performed to provide a graphical representation of DEMs having known biological relationships. Green icons indicate down-regulated miRNAs or genes and red icons indicates up-regulated miRNAs or genes. For each analysis, the most significant networks were selected and shown. In this figure we show the network analysis of the DEMs common to BEAS-PIK3CA-E545K, BEAS-AKT1-E17K and BEAS-shPTEN.

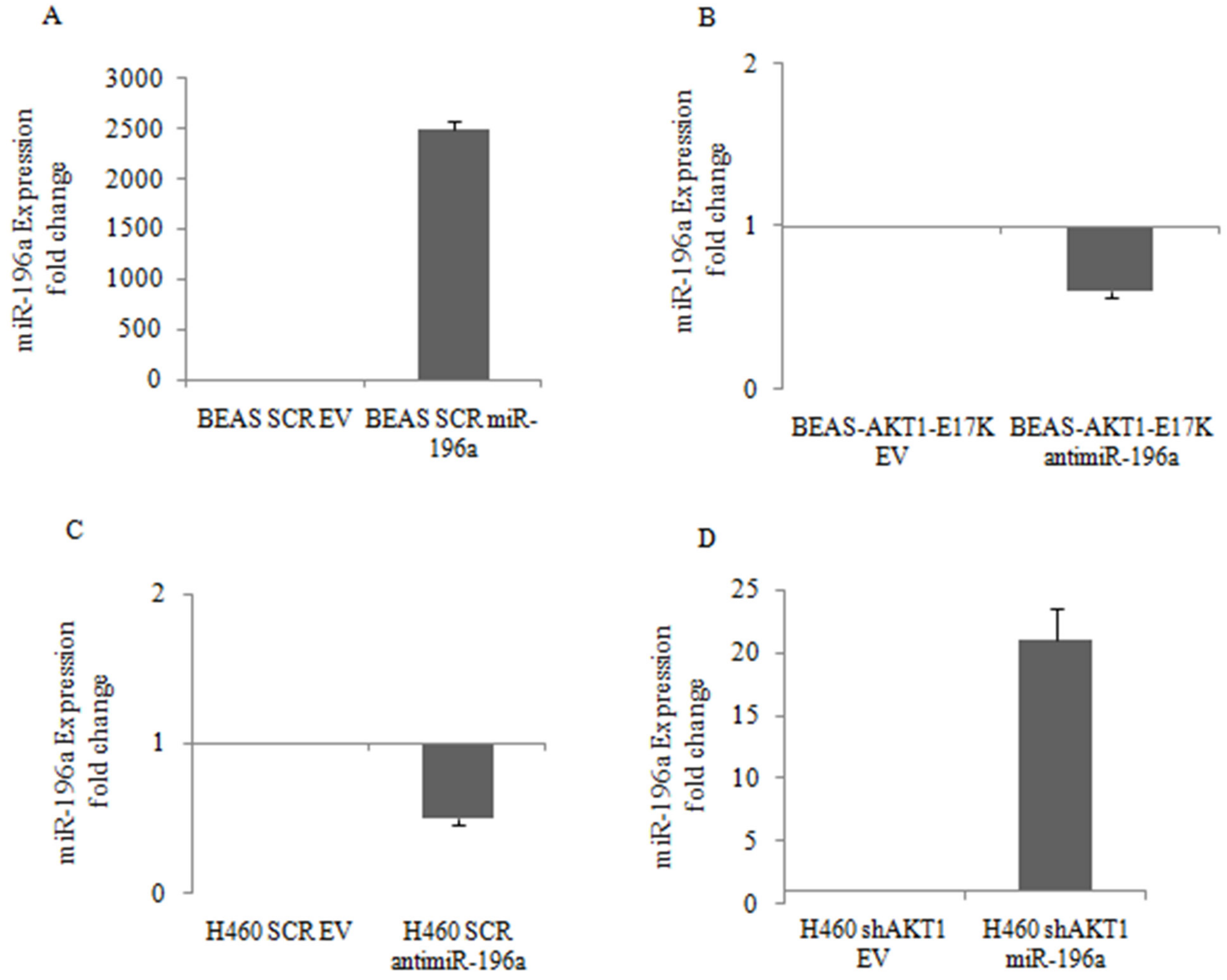

Supplementary Figure S3: qRT-PCR analysis of miR-196a. A.miR-196a expression levels in NCI-H460 cells transduced with control (EV) or antimiR-196a expressing lentiviruses. **B.**miR-196a expression levels in NCI-H460-shAKT1 cells transduced with control (EV) or miR-196a expressing lentiviruses. **C.** Expression of miR-196a in BEAS-2B cells transduced with control (EV) or miR-196a expressing lentiviruses. **D.** Expression of miR-196a in BEAS-AKT1-E17K cells transduced with control (EV) or antimiR-196a expressing lentiviruses. The relative expression values indicate the relative change in the expression levels between cell lines assuming that the value of control cells is equal to 1. Each bar represents the mean values ± SD from experiments performed in triplicate.

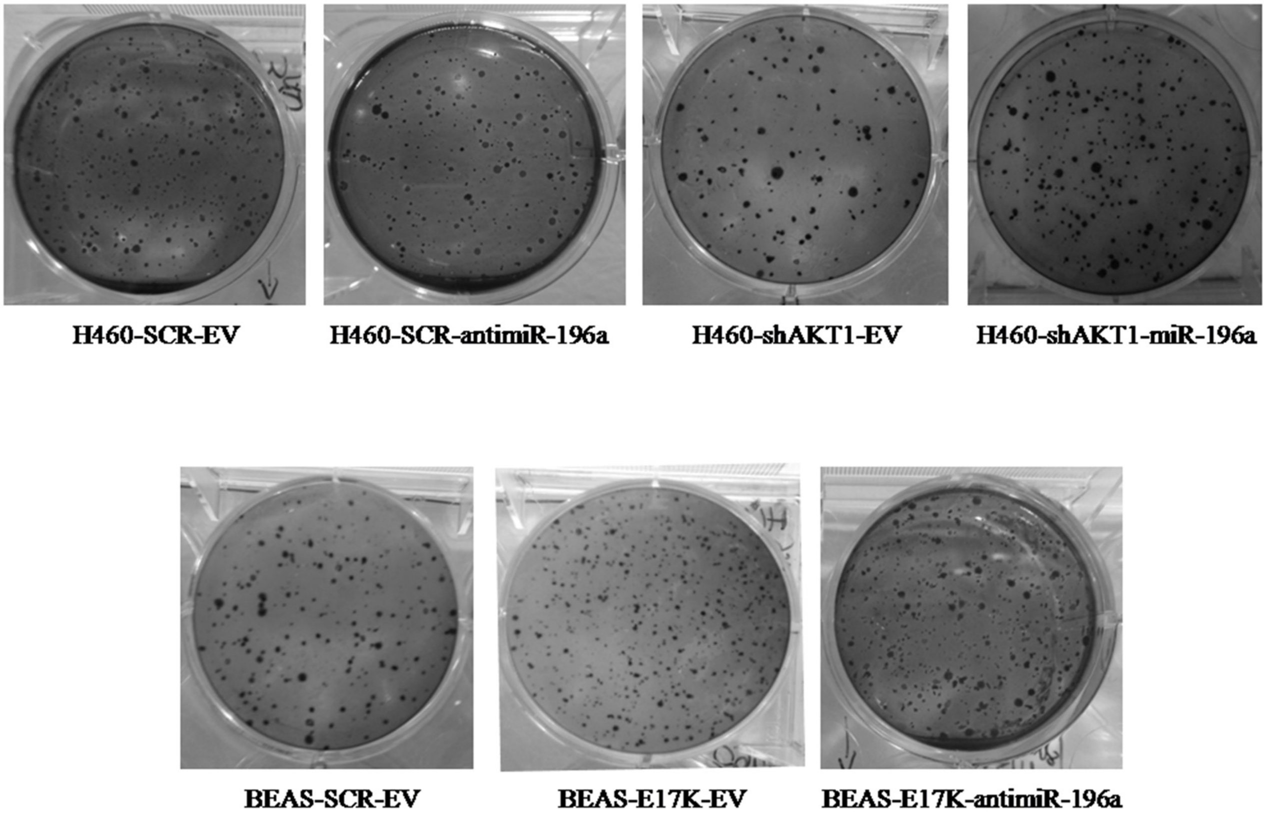

Supplementary Figure S4: Soft Agar assay. Representative images of soft agar colony formation assay.

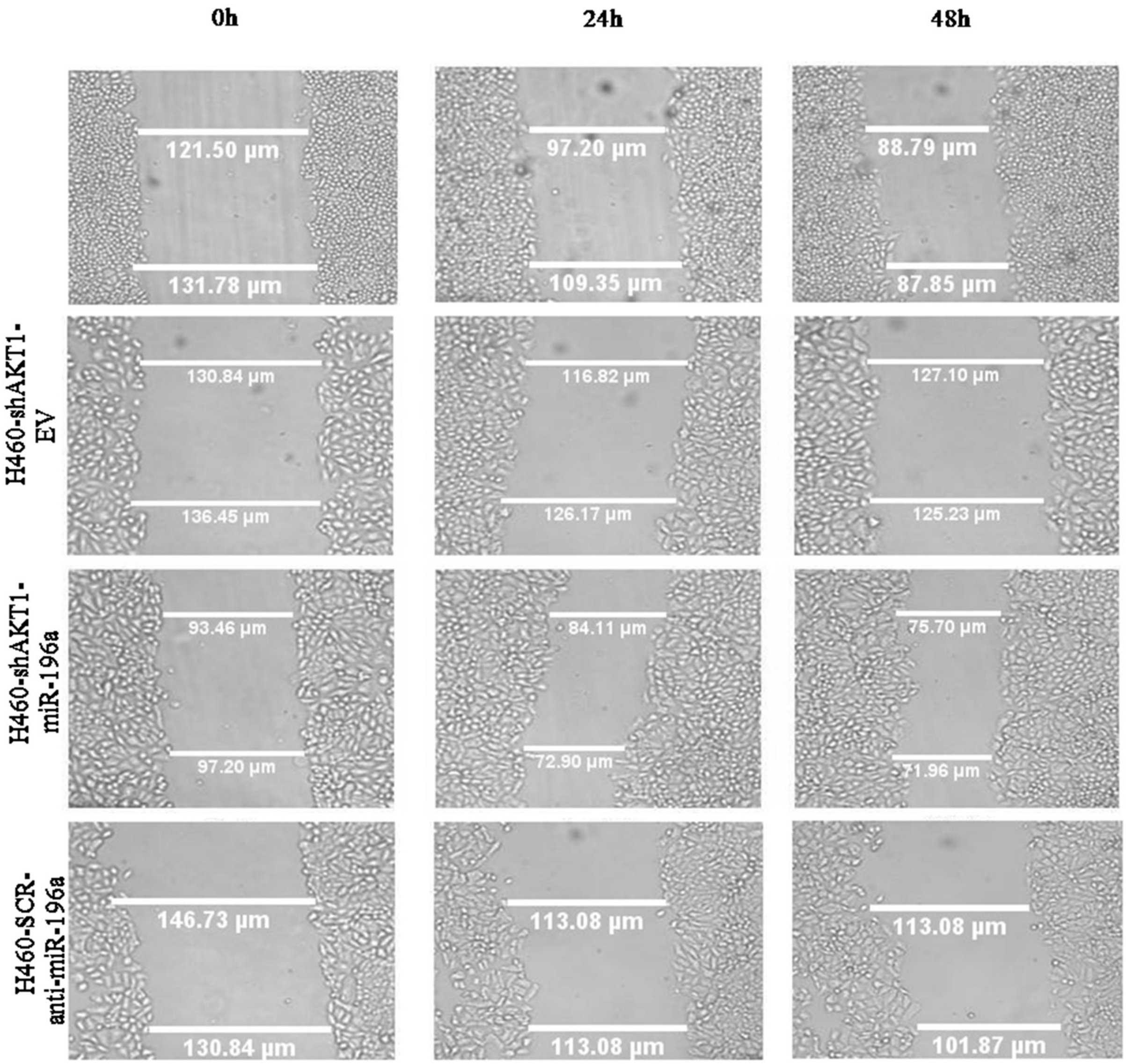

Supplementary Figure S5: Wound Healing assay. Representative images of wound closure after 24 and 48 hours from scratch.

Supplementary Table S1: MiRNAs significantly up-regulated or down-regulated in BEAS-AKT1-E17K compared with BEAS-SCR.

See Supplementary File 1

Supplementary Table S2: MiRNAs significantly up-regulated or down-regulated in BEAS-PIK3CA-E545K compared with BEAS-SCR.

See Supplementary File 2

Supplementary Table S3: MiRNAs significantly up-regulated or down-regulated in BEAS-shPTEN compared with BEAS-SCR.

See Supplementary File 3

Supplementary Table S4: DEMs showing the highest fold changes that were specific for mutant AKT1.

See Supplementary File 4

Supplementary Table S5: DEMs showing the highest fold changes that were specific for mutant PIK3CA.

See Supplementary File 5

Supplementary Table S6: DEMs showing the highest fold changes that were specific for PTEN loss.

See Supplementary File 6

Supplementary Table S7: A. Bio-functions identified by IPA in the DEMs common to BEAS-PIK3CA-E545K, BEAS-AKT1-E17K and BEAS-shPTEN. The list is organized on the basis of the B-H p-value**. B.** Sub-Categories and Functions identified through IPA showing the DEMs associated to lung cancer from BEAS-PIK3CA-E545K, BEAS-AKT1-E17K and BEAS-shPTEN.

See Supplementary File 7

Supplementary Table S8: List of Transcription Factors whose binding sites are present in the promoter region of miR-196a.

See Supplementary File 8
