# Supplementary material for: Analysis of miRNA profiles identified miR-196a as a crucial mediator of aberrant PI3K/AKT signaling in lung cancer cells
Source: Oncotarget. 2016 Nov 17;8(12):19172–91. doi: 10.18632/oncotarget.13432 (PMC5386676; doi:10.18632/oncotarget.13432)
Supplement: Supplementary file 8 [file oncotarget-08-19172-s008.doc]

| BIO-FUNCTIONS BEAS-PIK3CA-E545K vs BEAS-shPTEN vs BEAS-AKT1-E17K | | | |
| --- | --- | --- | --- |
| Category | Function | B-H-value | # Molecules |
| Cancer | cervical carcinoma | 2,63E-11 | 8 |
| Cancer | pancreatic cancer | 7,34E-08 | 8 |
| Cancer | uterine cancer | 1,34E-06 | 10 |
| Cancer | liver cancer | 1,32E-05 | 8 |
| Cancer | gastrointestinal tract cancer | 3,27E-05 | 11 |
| Cancer | colorectal cancer | 5,08E-05 | 10 |
| Cancer | hepatocellular carcinoma | 1,93E-04 | 6 |
| Cancer | hematological neoplasia | 2,26E-04 | 8 |
| Cancer | head and neck cancer | 2,94E-04 | 7 |
| Cancer | squamous-cell carcinoma | 3,14E-04 | 6 |
| Cancer | gastric cancer | 1,78E-03 | 4 |
| Cancer | melanoma | 3,08E-03 | 5 |
| Cancer | lung squamous cell carcinoma | 6,56E-03 | 2 |
| Cancer | genital tumor | 6,58E-03 | 7 |
| Cancer | small cell lung cancer | 8,93E-03 | 2 |
| Cancer | non-small cell lung cancer | 1,82E-02 | 3 |
| Cancer | carcinoma | 3,76E-02 | 14 |

**Table S7:** Bio-functions identified by IPA in the DEMs common to BEAS-PIK3CA-E545K, BEAS-AKT1-E17K and BEAS-shPTEN.

A

| BIO-FUNCTIONS BEAS-PIK3CA-E545K vs BEAS-shPTEN vs BEAS-AKT1-E17K | | | |
| --- | --- | --- | --- |
| Category | Function | p-value | Molecules |
| CANCER | LUNG SQUAMOUS CELL CARCINOMA | 6,56E-03 | miR-16-5p (and other miRNAs w/seed AGCAGCA),miR-203-3p (and other miRNAs w/seed UGAAAUG) |
|  |  |  |  |
|  | SMALL CELL LUNG CANCER | 8,93E-03 | miR-19b-3p (and other miRNAs w/seed GUGCAAA) |
|  |  |  |  |
|  | NON-SMALL CELL LUNG CANCER | 1,82E-02 | miR-16-5p (and other miRNAs w/seed AGCAGCA), miR-196a-5p (and other miRNAs w/seed AGGUAGU) |

B
