# Supplementary material for: Analysis of miRNA profiles identified miR-196a as a crucial mediator of aberrant PI3K/AKT signaling in lung cancer cells
Source: Oncotarget. 2016 Nov 17;8(12):19172–91. doi: 10.18632/oncotarget.13432 (PMC5386676; doi:10.18632/oncotarget.13432)
Supplement: Supplementary file 9 [file oncotarget-08-19172-s009.doc]

| TRANSCRIPTION FACTORS ON miR-196a PROMOTER | | | | | | |
| --- | --- | --- | --- | --- | --- | --- |
| GENE SYMBOL | **MATRIX_ID** | **Z_SCORE** | **P_VALUE** | **SAMPLE**  **AVERAGE** | **BACKGROUND**  **AVERAGE** | **SAMPLE**  **DEVSTD** |
| ESR1 | MA0112.2 | 2.682 | 0.004 | 0.851 | 0.750 | 0.851 |
| Rarb | MA0857.1 | 2.664 | 0.004 | 0.848 | 0.721 | 0.848 |
| ESR2 | MA0258.2 | 2.662 | 0.004 | 0.922 | 0.821 | 0.922 |
| E2F7 | MA0758.1 | 2.604 | 0.005 | 0.837 | 0.719 | 0.837 |
| TP53 | MA0106.1 | 2.379 | 0.009 | 0.803 | 0.724 | 0.803 |
| NHLH1 | MA0048.1 | 2.361 | 0.009 | 0.949 | 0.841 | 0.949 |
| HINFP | MA0131.1 | 2.224 | 0.013 | 0.921 | 0.827 | 0.921 |
| NHLH1 | MA0048.2 | 2.221 | 0.013 | 0.960 | 0.849 | 0.960 |
| Rarg | MA0859.1 | 2.161 | 0.015 | 0.853 | 0.743 | 0.853 |
| Gata1 | MA0035.3 | 2.150 | 0.016 | 0.957 | 0.852 | 0.957 |
| Nr2f6(var.2) | MA0728.1 | 2.132 | 0.016 | 0.862 | 0.751 | 0.862 |
| MYF6 | MA0667.1 | 2.083 | 0.019 | 0.922 | 0.822 | 0.922 |
| GCM2 | MA0767.1 | 2.059 | 0.020 | 0.934 | 0.855 | 0.934 |
| MAFF | MA0495.1 | 1.965 | 0.025 | 0.868 | 0.792 | 0.868 |
| RARA | MA0729.1 | 1.947 | 0.026 | 0.810 | 0.728 | 0.810 |
| Vdr | MA0693.1 | 1.938 | 0.026 | 0.826 | 0.757 | 0.826 |
| SPIC | MA0687.1 | 1.935 | 0.027 | 0.906 | 0.830 | 0.906 |
| CENPB | MA0637.1 | 1.912 | 0.028 | 0.863 | 0.799 | 0.863 |
| Gata1 | MA0035.2 | 1.849 | 0.032 | 0.969 | 0.883 | 0.969 |
| GATA3 | MA0037.2 | 1.802 | 0.036 | 0.974 | 0.874 | 0.974 |
| GATA2 | MA0036.2 | 1.800 | 0.036 | 0.922 | 0.843 | 0.922 |
| ESR1 | MA0112.1 | 1.799 | 0.036 | 0.866 | 0.804 | 0.866 |
| TP53 | MA0106.3 | 1.788 | 0.037 | 0.796 | 0.732 | 0.796 |
| TP63 | MA0525.1 | 1.762 | 0.039 | 0.865 | 0.803 | 0.865 |
| RORA(var.2) | MA0072.1 | 1.759 | 0.039 | 0.884 | 0.802 | 0.884 |
| Esrrb | MA0141.2 | 1.725 | 0.042 | 0.933 | 0.852 | 0.933 |
| Esrrb | MA0141.1 | 1.716 | 0.043 | 0.933 | 0.852 | 0.933 |
| YY1 | MA0095.2 | 1.701 | 0.044 | 0.908 | 0.825 | 0.908 |
| MAFK | MA0496.1 | 1.695 | 0.045 | 0.898 | 0.826 | 0.898 |
| Foxq1 | MA0040.1 | 1.601 | 0.055 | 0.940 | 0.853 | 0.940 |
| RORA | MA0071.1 | 1.570 | 0.058 | 0.937 | 0.861 | 0.937 |
| Crx | MA0467.1 | 1.568 | 0.058 | 0.914 | 0.849 | 0.914 |
| CREB3 | MA0638.1 | 1.558 | 0.060 | 0.843 | 0.780 | 0.843 |
| Bcl6 | MA0463.1 | 1.547 | 0.061 | 0.887 | 0.823 | 0.887 |
| E2F8 | MA0865.1 | 1.537 | 0.062 | 0.826 | 0.757 | 0.826 |
| Znf423 | MA0116.1 | 1.512 | 0.065 | 0.907 | 0.847 | 0.907 |
| XBP1 | MA0844.1 | 1.496 | 0.067 | 0.834 | 0.775 | 0.834 |
| PRDM1 | MA0508.1 | 1.455 | 0.073 | 0.877 | 0.818 | 0.877 |
| Tcf3 | MA0522.1 | 1.448 | 0.074 | 0.962 | 0.895 | 0.962 |
| TP53 | MA0106.2 | 1.438 | 0.075 | 0.835 | 0.781 | 0.835 |
| EHF | MA0598.1 | 1.418 | 0.078 | 1.000 | 0.916 | 1.000 |
| RFX5 | MA0510.1 | 1.405 | 0.080 | 0.906 | 0.858 | 0.906 |
| Gata4 | MA0482.1 | 1.403 | 0.080 | 0.939 | 0.880 | 0.939 |
| NFATC2 | MA0152.1 | 1.400 | 0.081 | 1.000 | 0.937 | 1.000 |
| GCM1 | MA0646.1 | 1.400 | 0.081 | 0.877 | 0.824 | 0.877 |
| SPI1 | MA0080.4 | 1.399 | 0.081 | 0.833 | 0.770 | 0.833 |
| MZF1(var.2) | MA0057.1 | 1.398 | 0.081 | 0.973 | 0.928 | 0.973 |
| MGA | MA0801.1 | 1.393 | 0.082 | 0.961 | 0.893 | 0.961 |
| Tal1::Gata1 | MA0140.1 | 1.386 | 0.083 | 0.871 | 0.814 | 0.871 |
| TCF3 | MA0522.2 | 1.365 | 0.086 | 0.988 | 0.919 | 0.988 |
| Esrrg | MA0643.1 | 1.355 | 0.088 | 0.916 | 0.851 | 0.916 |
